# Reinfection or Reactivation of Severe Acute Respiratory Syndrome Coronavirus 2: A Systematic Review

**DOI:** 10.3389/fpubh.2021.663045

**Published:** 2021-06-11

**Authors:** Xiujuan Tang, Salihu S. Musa, Shi Zhao, Daihai He

**Affiliations:** ^1^Shenzhen Center for Disease Control and Prevention, Shenzhen, China; ^2^Department of Applied Mathematics, The Hong Kong Polytechnic University, Hong Kong, China; ^3^Department of Mathematics, Kano University of Science and Technology, Wudil, Nigeria; ^4^Jockey Club School of Public Health and Primary Care, Chinese University of Hong Kong, Hong Kong, China; ^5^Shenzhen Research Institute of Chinese University of Hong Kong, Shenzhen, China

**Keywords:** COVID-19, pandemic, re-detectable positive, reactivation, reinfection

## Abstract

As the pandemic continues, individuals with re-detectable positive (RP) SARS-CoV-2 viral RNA among recovered COVID-19 patients have raised public health concerns. It is imperative to investigate whether the cases with re-detectable positive (RP) SARS-CoV-2 might cause severe infection to the vulnerable population. In this work, we conducted a systematic review of recent literature to investigate reactivation and reinfection among the discharged COVID-19 patients that are found positive again. Our study, consisting more than a total of 113,715 patients, indicates that the RP-SARS-CoV-2 scenario occurs plausibly due to reactivation, reinfection, viral shedding, or testing errors. Nonetheless, we observe that previously infected individuals have significantly lower risk of being infected for the second time, indicating that reactivation or reinfection of SARS-CoV-2 likely have relatively less impact in the general population than the primary infection.

## Introduction

Severe acute respiratory syndrome coronavirus 2 (SARS-CoV-2) is a respiratory virus from the family *coronaviradae* and order *nidovirales*. Other viruses from the same family include the severe acute respiratory syndrome coronavirus (SARS-CoV) and middle east respiratory syndrome coronavirus (MERS-CoV), which are known to infect humans and caused hundreds of thousands of deaths worldwide (mostly in Asia and the middle east) (1, 2). COVID-19 is caused by SARS-CoV-2 and has been considered a devastating public health problem globally since its emergence in China in late 2019. The disease has, as of 29 March 2021 affected about 127 million people with over 2.7 million deaths across the globe (3). Due to effective and timely interventions, more than 70 million people have already recovered from the SARS-CoV-2 infection, indicating the impact of timely interventions and treatment which showed remarkable progress by facilitating the recovery of large number of patients even before the emergence of vaccines against the infection (3–5).

Recently, the issue of reinfection (SARS-CoV-2 subsequent infection after recovery from previous episode of the infection) and reactivation (also known as relapse, a re-detectable positive SARS-CoV-2 viral RNA in recovered patient which occurs within the first 4 weeks of previous infection) have been reported in several studies [see for instance (6–21), and the references therein]. These studies highlighted the possibility of reactivation and reinfection of SARS-CoV-2 which needs urgent attention from the researchers as well the public health policymakers. A re-detectable positive (RP) SARS-CoV-2 infection is ascertained commonly by using reverse transcriptase-polymerase chain reaction (RT-PCR) test from a COVID-19 patient after recovery from the primary infection before confirmation of reactivation or reinfection. Moreover, the positivity of RT-PCR can also be detected due to RNA viral shedding (22) or diagnostic testing errors likely due to technical issues of RT-PCR assays (23). A recent retrospective study by Agarwal et al. (22) who analyzed 851 SARS-CoV-2-positive patients with at least two positive PCR tests found that 99 of them remained SARS-CoV-2-positive after 28 days from their initial diagnosis date. The report showed that the median lower and upper bounds for viral RNA shedding in COVID-19 patients occurred between 2 to 3 weeks (22).

This raises serious concerns on whether a more precautionary measures should be considered in declaring the recovery phase from COVID-19 infection, and the significance of follow-up, especially in the most vulnerable population (24). In this work, we reviewed some primary studies that evaluated the possible reactivation and/or reinfection of SARS-CoV-2 mostly based on clinical or laboratory reports to shed more light on possible reactivation and/or reinfection of SARS-CoV-2 by recovered patients after satisfying the standard discharge criteria. Our study aimed to provide recommendations to help to prevent further spread of the virus since most clinical features, significance, and the potential cause of RP-SARS-CoV-2 patients remain unclear.

### Standard Discharge Criteria for SARS-CoV-2 Patients

The standard discharge criteria from the isolation/hospitalization process for a COVID-19 patient who recovered from a primary episode of the infection (4, 5, 9, 10, 25–28) are summarized as follows:

1. Normal temperature (<38°C) for more than 72 h consecutively before the discharge;
2. A notable improvement in respiratory symptoms;
3. Clear acute exudative lesions of chest computed tomography (CT) images must be improved;
4. Two consecutive negative results for RT-PCR carried out at least 24-h apart;
5. Hospital care no longer required;
6. Specific IgG appearance by a serological test.

According to previous studies (9–11), some COVID-19 patients were found positive from RT-PCR results for the second time (usually) within 5–13 days after discharge from the isolation before confirmation of reactivation or relapse (5, 29, 30), while some patients were found to be RP-SAR-CoV-2 at least 4 weeks from the first episode of the infection, indicating the possibility of reinfection (6, 7). Therefore, urgent research is needed to disentangle possible reasons of RP- SAR-CoV-2 after recovery from primary infection to guide policy-making and help in controlling further spared of the virus (9, 31–34).

Currently, there is little knowledge or information about possible reasons for RP-SARS-CoV-2, which might probably be due to reactivation, reinfection, viral shedding, or testing errors. Nonetheless, many reports on possible reactivation and reinfection in recovered COVID-19 patients were asymptomatic or have mild to moderate symptoms (27, 35) which typically recover within 14 days interval. Table 1 summarizes the characteristics of RP-SARS-CoV-2 in recovered patients, which occurs plausibly due to reactivation or reinfection, including the population-based observational study in Denmark consisting of 4 million individuals with possible reinfection of 2.11% (16).

**Table 1:** Recurrence of SARS-CoV-2 in recovered patients.

**Country**	**Age (years)**	**Gender (among RP)**	**Number of patient involve in study**	**Symptoms on first infection**	**Symptoms on second infection**	Time interval between discharged and RP (days)^*^	**Rate of infection**	**Remark**	**References**
Hong Kong, China	33	M	1	Symptomatic	Asymptomatic	123		Reinfection	(6)
USA	25	M	1	Symptomatic	Symptomatic with hospitalization	48		Reinfection	(36)
Belgium	52	F	1	Symptomatic	Symptomatic	93		Reinfection	(37)
Ecuador	46	M	1	Symptomatic	Symptomatic	63		Reinfection	(38)
India	25% 28	F = 1, M = 1	2	Asymptomatic	Asymptomatic	100 and 101		Reinfection	(39)
China	46	F = 1	1	Mild	Mild	6		Reactivation	(40)
Mexico	At least 20	F = 53.9% among reinfection	100,432	Asymptomatic or mild to severe	Mild to severe	28	258/100432 = 0.26%	Reinfection	(7)
China	30–36	F = 2; M = 2	4	3 Mild to moderate, and 1 asymptomatic	Asymptomatic	5–13		Reactivation	(9)
China	27–89 (Median age = 56)	F = 12; M = 11	651	Mild to moderate	12 = moderate, 9 = severe, and 2 = critical	Median = 15	23/651 = 3%	Reactivation	(10)
China		F>M	209	Mild to moderate	Mild to moderate	2–13	22/209 = 10.5%	Reactivation	(11)
China	<60		262	Mild to moderate and severe	Mild to moderate	14	38/262 = 14.5%	Reactivation	(12)
USA	82	M	1	Mild to moderate	Mild to moderate	10		Reactivation	(31)
USA			1			28		Reinfection	(22)
China	47.0 (40.5–55.5)	F = 9 (35%), M = 16 (64%)	51	Mild to moderate and severe	Mild to moderate and severe	12–26		Reactivation	(41)
Turkey	46 and 47	M	2	Mild	Mild	100 and 104		Reinfection	(42)
China	12–49	F = 2, M = 2	17	Mild to moderate	Mild	3		Reactivation	(43)
USA	55	F	1	Mild	Mild to moderate	18		Reactivation	(44)
China	50	M	1	Mild to moderate	Mild to moderate	40		Reinfection	(45)
China	1–73	F = 7, M = 6	13	Mild to moderate	Mild to moderate	5–14	31% (4/13)	Reactivation	(46)
China	57	F = 1	1	Mild to moderate	Mild to moderate	4		Reactivation	(47)
China	0.92–86		92	Mild to moderate and severe	Mild to moderate and severe	2–48			(48)
China	0.25–69	F = 42,M = 45		Mild to moderate	Mild to moderate	2–19		Reactivation	(23)
China	2.5–12.7	F = 13, M = 11		Mild to moderate	Mild to moderate	2.4–12		Reactivation	(49)
China	4–80 (Median age = 37.2)	F = 8,M = 12	147	Mild to moderate	Mild to moderate	Median = 17.25		Reactivation	(50)
China	33.5–58.5 (Median age = 46.5)	F = 26,M = 34		Mild to moderate	Mild to moderate	4–24		Reactivation	(51)
China	Median age = 34	F = 57,M = 36				7–14		Reactivation	(52)
China				Mild to moderate	Mild to moderate	7–11		Reactivation	(29)
China	2–7		14			7–17		Reactivation	(53)
China	18–90 (Median age = 48)	F = 157,M = 128	285	Mild to moderate and severe	Mild to moderate and severe	5–8	F = 65.6, M = 44.4	Reactivation	(54)
China	40	M	1	Mild to moderate and severe	Mild to moderate	5		Reactivation	(55)
China	Median age = 54	F = 70.6%,M = 29.4%	98	Mild to moderate	Mild to moderate	<17	F = 5/32,M = 12/66	Reactivation	(56)
France	19–91	F = 45.5%,M = 54.5%		Mild to severe	Mild to severe	4–27		Reactivation	(57)
China	60–76	M = 33.3%	126		Asymptomatic	10–18		Reactivation	(58)
Korea	0–>80		8922		Asymptomatic to mild	Median = 19		Reactivation	(59, 60)
China	<29–79	F = 59%,M = 41%	576			Median = 14		Reactivation	(61)
China	1–72	M = 13(65%)	182		Mild to moderate	7–14		Reactivation	(62)
China	<12–60	M = 25 (14.5%)	172	Mild to severe	Mild to moderate	3.46–11.18		Reactivation	(63)
China	Range = 23–68	M = 4 (26.7%)	85	Mild to moderate	Mild to moderate	9–30			(64)
Iran	Median = 52	M = 5 (55.6%)	13	Mild to moderate	Mild to moderate	15–48			(65)
China	26–72	M = 3 (37.5%)	108	Mild to severe	Asymptomatic	6–28		Reactivation	(66)
China	36–66	M = 4 (66.7%)	11		Mild to moderate	6–27		Reactivation	(67)
China		M = 10 (40%)	68	Mild to moderate		<7		Reactivation	(68)
China		M = 9 (17.6%)	51		Mild to moderate	7–14		Reactivation	(69)
China	9–62	M = 8 (53.3%)	15	Moderate	Mild	15		Reactivation	(70)
Brunei Darussalam	Median = 47	M = 12 (57.1%)	106		Asymptomatic and mild	11–17		Reactivation	(71)
China		M = 1 (50%)	62	Mild	Asymptomatic	6–14		Reactivation	(72)
China	23–57	M = 14 (70%)	20	Mild	Asymptomatic	7		Reactivation	(73)
China	29–87	M = 23 (43.4%)	257	Mild to severe	Asymptomatic and mild	1–12		Reactivation	(74)
China		M = 12 (54.5%)	161			1–14		Reactivation	(75)
China			37			1–6		Reactivation	(76)
China	27–42	M = 2 (40%)	55		Mild to moderate	4–17		Reactivation	(77)
Italy	37–78	M = 3 (50%)	29	Mild to moderate	Asymptomatic	13–24		Reactivation	(78)
China	18–71	M = 12 (63.2%)	71	Mild to severe	Mild to severe	1–17		Reactivation	(79)
China	19–79	M = 12 (44.4%)	285		Asymptomatic	15		Reactivation	(73)
China	34			Severe	Asymptomatic	15		Reactivation	(80)
China	34–74	M = 1 (33.3%)		Mild	Asymptomatic	1–5		Reactivation	(81)
China	70	M = 1	1	Moderate to severe	Asymptomatic	13		Reactivation	(82)
China	35	M = 1	1	Mild	Mild	14			(83)
Italy	48	M = 1	1	Severe	Moderate	30		Reinfection	(84)
China	<67	M = 4 (57.1%)		Mild to moderate	Asymptomatic	7–13		Reactivation	(85)
China	54	M = 1	1	Moderate	Asymptomatic	4		Reactivation	(86)
Brazil	26	M = 1	1	Mild	Severe	30		Reinfection	(87)
China	21 and 55	M = 2	2	Moderate		17		Reactivation	(88)
China	30–56				Mild to moderate	3–14		Reactivation	(89)
China	8	M = 1	1	Mild	Mild	15		Reactivation	(90)
Korea	8	M = 1	1	Mild	Mild	14		Reactivation	(91)
Switzerland	77 and 81	F = 2	2	Moderate	Moderate and severe	14–21		Reactivation	(34)
Italy	69	F = 1	1	Mild to moderate	Asymptomatic	23		Reactivation	(92)
Korea	72	F = 1	1	Moderate		6		Reactivation	(93)

* *Time interval between discharged and RP represents the period between the discharge and the time during which a patient tested positive again. Gender represents the RP of each gender*.

## Methods

### Searching Strategy and Study Screening Process

We conducted a systematic review on the possibilities of reactivation and reinfection of SARS-CoV-2 in recovered patients that covered published peer-reviewed articles in the literature from Nov 1, 2019, to Mar 29, 2021. Following the guidelines by the “Preferred Reporting Items for Systematic reviews and meta-Analyses” (PRISMA) (34), we searched the following databases: MEDLINE; PubMed; and Embase for papers published in English, among which only human participants were studied. Our search strategy includes (severe acute respiratory syndrome coronavirus 2 OR SARS-CoV-2 OR 2019-nCoV OR coronavirus disease 2019 OR COVID-19) AND (reinfection OR reactivation OR relapse OR RNA shedding OR viral shedding OR re-detectable positive) AND (recovered patients OR discharge patients OR post-COVID-19 patients). Related references were also searched through preprint servers (bioRxiv and medRxiv) and general google search, and reviewing the reference list of the included articles. Letters to editors and commentaries were also included to ensure robust coverage of the existing literature. All retrieved records were imported into the ENDNOTE citation software and duplicates were removed using the ENDNOTE built-in “Find Duplicates” feature. Finally, the titles, abstracts, and full text of the generated studies were sequentially screened to ascertain the studies that met the inclusion criteria of the review.

### Study Selection and Eligibility Criteria

**Figure 1:**
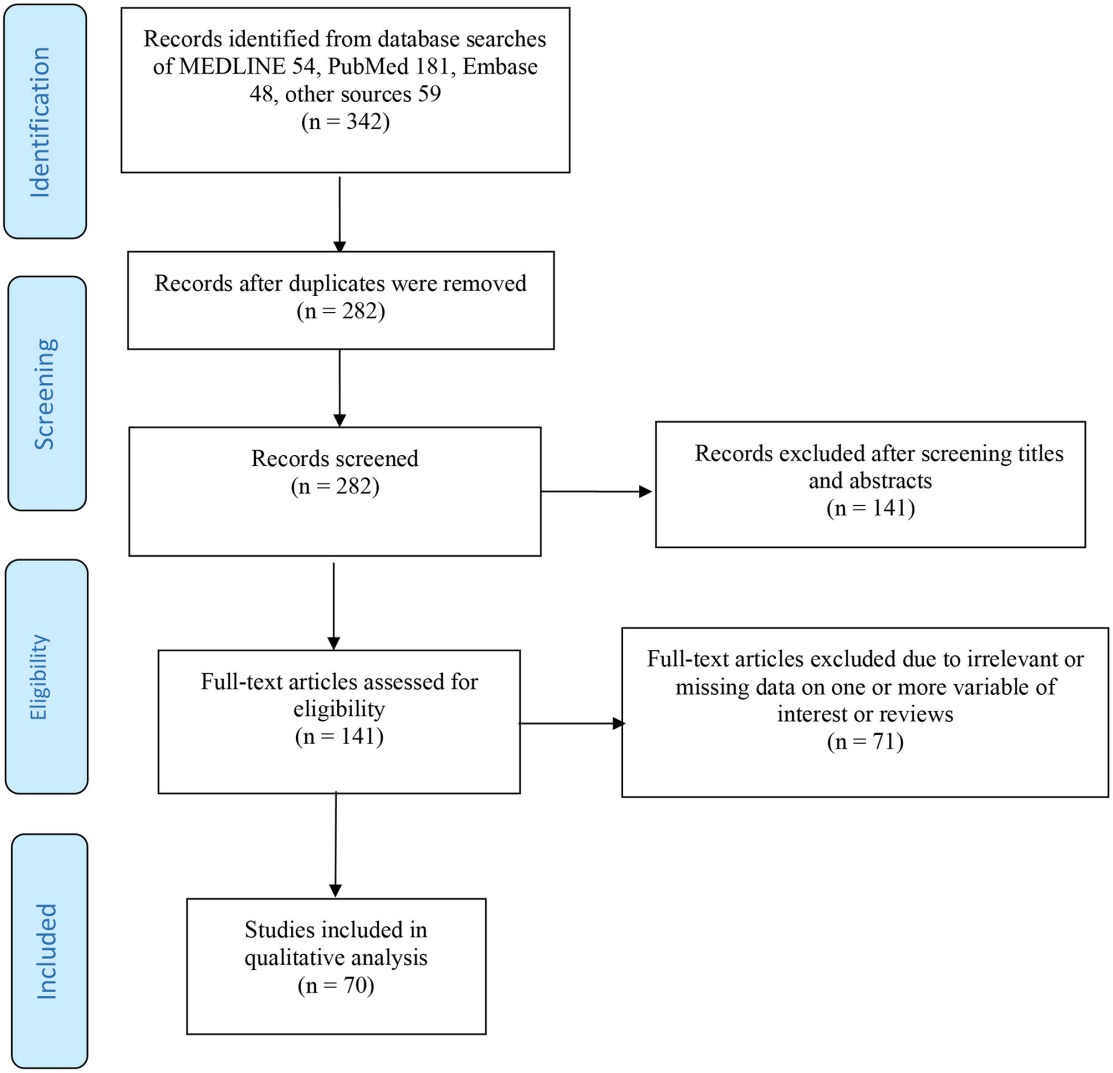
Flow diagram of the searching strategy and article selection process.

The following inclusion criteria were used in study selection: (i) articles published in peer-reviewed journals, case reports, letters to editors, and commentaries; (ii) articles studying the COVID-19 reactivation or reinfection in recovered patients; as well as SARS-CoV-2 RNA shedding in recovered COVID-19 patients; and (iii) articles published in English or at least with an abstract in the English language. A flow chart of the search strategy and study selection process is presented in Figure 1 using PRISMA guidelines (94). Studies that reported the possibility of reactivation and/or reinfection of SARS-CoV-2 for patients with other comorbid conditions such as asthma, old age, and type 2 diabetes were also included in this study. It is important to note that the following exclusion criteria were used in this study: (i) studies with irrelevant topics; (ii) lack of information (data) or ineligible article types; (iii) review studies; and (iv) review protocol. Similarly, research articles reported SARS-CoV-2 reactivation or reinfection in recovered patients published in a non-English language, or have no accessible full-text access were also excluded. Study search and screening processes were conducted independently by two reviewers/authors (SSM and SZ).

### Data Extraction and Analysis

Relevant data were extracted independently by SSM and SZ to ensure accurate reporting. The generated results were compared, and any inconsistency in the data was resolved by further discussions among the authors. The generated results were then further synthesized. The data analyzed included the incidence of SARS-Cov-2 reactivation and reinfection in recovered COVID-19 patients.

## Results

### Search Findings

In total, 342 articles were identified in total (54, MEDLINE; 181, PubMed; 48, Embase; and 59, other sources). There were 282 studies left after removing the duplicates. After 141 articles were excluded by screening the titles and abstracts, we retrieved 141 articles eligible for the full-text screening. We excluded 71 articles based on the aforementioned exclusion criteria. Eventually, 70 studies that satisfied the eligibility criteria were included in this review for further analyses, and they include primary research studies, letters to editors, commentaries, and case reports. Overall, the included studies recruited more than 113,715 patients.

### Epidemiological Findings

The reviewed studies covered SARS-CoV-2 incidence of possible reactivation, reinfection, or viral shedding worldwide. The majority of the studies were from China. Subsequently, the included studies (Table 1) estimated the time interval for RP-SARS-CoV-2 in recovered patients after follow-up, following discharge from isolation, or hospitalization after satisfying standard discharge criteria.

#### RP-SARS-CoV-2 Reinfection Possibilities

Following previous reports (6, 7, 12, 32), we re-examined some of the clinical features, infection ratios, recovery, and potential reasons of possible reactivation and/or reinfection of SARS-CoV-2 to shade more light on the current issue of RP-SARS-CoV-2 in recovered patients, and provide suggestions for public health policy-makers to guide effective control of SARS-CoV-2 transmission. Our study would be valuable to policy-makers since there was until recently no clear epidemiological underpinning explanation for the resurgence of COVID-19 infection among patients that tested positive on a retest.

Here, we reported some scenarios that analyze possible reinfection of SARS-CoV-2 in recovered patients. A recent retrospective cohort study in Mexico by Murillo-Zamora et al. (7) revealed some possible factors that predict severe symptomatic SARS-COV-2 reinfection, which suggested that reinfection occurs when the time lag between discharge and RP is at least 28 days (that is, a second-time infection after a patient satisfied the standard discharge criteria). Moreover, they found that the risk of previously infected patients being infected for the second time was 258/100,432 = 0.26%, with a case fatality rate of 11/258 = 4.3%, while the overall infection attack rate in Mexico, as of 18 January 2021, was 1.273% with a case fatality rate as 8.572%. Note that, as of November 17, 2020, Mexico has 1,641,428 COVID-19 cases, including 140,704 associated deaths (3, 95). Their results also revealed some multiple factors related to an increased risk of severe symptomatic SARS-COV-2 reinfection, which was asthma 1.26 (95% CI: 1.06–1.50), older age 1.007 (95% CI: 1.003–1.010), obesity 1.12 (95% CI: 1.01–1.24), type 2 diabetes mellitus 1.22 (95% CI: 1.07–1.38), and previous severe SARS-CoV-2 infection 1.20 (95% CI: 1.03–1.39).

Another recent clinical study by Duggan et al. (31) examined an 82-year-old COVID-19 patient who has been identified with some underline health conditions (including a history of advanced Parkinson's disease, insulin-dependent diabetes, chronic kidney disease, and hypertension). After recovery, the patient tested positive to SARS-CoV-2 on a re-test at least 48 days after the first infection, indicating that the RP was likely due to reinfection. Also, a study by To et al. (6) in Hong Kong reported a situation of RP-SARS-CoV-2 by a 33-year-old man that was detected 123 days after the previous episode of the infection (following discharge). During the period of the first infection, the symptoms were mostly mild, which was resolved/improved during the isolation or hospitalization process. A total of 2 weeks later, the patient satisfied the standard discharge criteria and was discharged from the hospital, following two consecutive negative SARS-CoV-2 results carried out by RT-PCR test at least 24-h apart. The second infection was detected and found to be asymptomatic but a different strain from the previous episode. This showed that the RP differs from the first infection (strain) which was verified by whole-genome analysis. The two strains belonged to different origin or clades with 24 nucleotide differences, which was of high quantity considering the relatively slow mutation rate detected for SARS-CoV-2 up-to-date. The first strain identified has a similar origin to the viruses that originated from Hong Kong, while the second strain identified has a similar origin to viruses from Spain. Consequently, another useful way to detect the positivity of RT-PCR is due to viral shedding from previous infections (22).

#### Possible Relapse Rather Than Reinfection of SARS-CoV-2

In this section, we reported scenarios that analyzed the possible reactivation of SARS-CoV-2 in recovered patients. According to a clinical report by Lan et al. (9), four medical workers aged 30–36 years old were found to be RP-SARS-CoV-2 within 5–13 days from recovery from the first episode of the infection. The patients were discharged from the isolation following the standard discharge procedure (9). This highlighted that some recovered COVID-19 patients can still be positive (or carriers) on a retest. This draws wide attention and raises a lot of public health concerns. Moreover, a study by Mei et al. (10) showed that 23 of 651 patients (about 3%) who satisfied the standard discharge criteria tested positive on a retest during the follow-up processes after recovery from the first infection. The median age of the RP group was 56 years, and there were slightly more women than men. Thus, we observed that the average duration from discharge to subsequent infection within 15 days is more likely to be reactivation.

Furthermore, a study by Tang et al. (11) carried out in Shenzhen, China, re-examined 209 patients that recovered from COVID-19 infection following the standard discharge criteria. After follow-up, they found that 22 of the patients (about 10.5%) were RP for SARS-CoV-2 on a retest, highlighting a possibility of relapse, as the second time (RT-PCR) results were found to be positive at the interval of 2–13 days between discharge and subsequent infection (re-positive on re-test).

## Discussion

It has been more than a year since the COVID-19 pandemic started in Wuhan, China, and rapidly spread across the globe. Although a large portion of COVID-19 patients has gradually recovered, it is imperative to follow up with recovered patients to investigate possible reasons for RP-SARS-CoV-2. There are still a lot of unknown clinical features related to COVID-19 epidemiology, especially in recovered patients. In this regard, it is necessary to understand the epidemiological features of RP-SARS-CoV-2 in recovered patients and to examine whether they are potential threats to public health (96). Several studies that reported the situations of RP-SARS-CoV-2 suggested that subsequent infection mostly occurs due to reactivation or reinfection rather than testing errors or prolonged viral shedding (16). However, this issue needs urgent attention to investigate whether RP-SARS-CoV-2 patients could be a serious public health problem. However, some studies reported that a small proportion (about 1%) of the population can be RP for SARS-CoV-2, and possibly due to reactivation or reinfection (16, 17). Furthermore, previous reports (7, 34, 35) highlighted that RP-SARS-CoV-2 is less likely to cause serious problems to public health since the rate of RP seems low (about 1%), and new infections declining after recovery from the first episode of the infection (16, 35), which is likely due to the suspected herd immunity (97, 98). This suggests that previously infected individuals have a significantly lower risk of being infected for the second time. Consequently, the aforementioned studies highlighted the possibility of reactivation and/or reinfection of SARS-CoV-2, which is less likely to cause a serious public health problem. However, we argue that these issues of RP-SARS-CoV-2 need further investigation, even though a small proportion has been reported to be RP after discharge. This is due to the fact that, despite numerous studies on COVID-19 as part of the efforts to curtail the spread of the virus, up to date, a lot of its epidemiological features remained unknown.

Overall, we observed that (i) if the time lag between discharge and RP of SRAS-CoV-2 is at most 28 days, these might be reinfection or relapse of previous infection; (ii) if the time lag is 2 months, it is more likely to be reinfection; and (iii) if the time lag is 3 months or above, it is very likely to be true reinfection (17). However, the most reliable way is to perform sequencing twice and get two different strains of the virus. Also, a possible reactivation usually occurs when the time lag is at most 15 days following discharge from the first episode of the infection (9, 11). It is worth mentioning that the reactivation of SARS-CoV-2 by RT-PCR found at least 28 days were associated with substantial genetic differences. We also observed that few infected individuals were able to generate second-time infection following the RP-SARS-CoV-2, which is regarded as possible reactivation or reinfection (depending on the period for subsequent infection), and this can be identified using a RT-PCR test.

However, we emphasized that caution should be exercised especially for vulnerable populations even after recovery from SARS-CoV-2. Also, close monitoring on an outpatient basis appears crucial, since the clinical features and potential reasons for possible reactivation and reinfection remained unclear. Like other studies, our work is not free from limitations; for instance, the time interval to remark on a possible reason for RP of SARS-CoV-2 is short considering the emergence of the new COVID-19 strain in some parts of the world, and this may cause exclusion in some reinfected group of individuals. Thus, further studies should be done as more COVID-19 data is being collected worldwide.

## Conclusion

In this work, we reported the plausibility of SARS-CoV-2 reactivation and reinfection in the context of the growing body of literature surrounding the dynamic of SARS-CoV-2 using RT-PCR test results. Our findings suggested the importance of dynamic surveillance of SARS-CoV-2 viral RNA for infectivity examination or assessment. Although there is currently no clear evidence that the RP-SARS-CoV-2 patient causes severe infection in a vulnerable population, more precautionary measures should be taken in declaring recovery from COVID-19 infections. Our study also emphasized the importance of follow-up in recovered patients to prevent further spread of the virus. Finally, we found that previously infected individuals have a significantly lower risk of being infected again than the first time infection.

## Author Contributions

XT, SSM, and DH: conceptualization. XT, SSM, SZ, and DH: formal analysis and writing - original draft. DH: writing - review & editing. All authors listed have made a substantial, direct and intellectual contribution to the work, and approved it for publication.

## Conflict of Interest

DH was supported by an Alibaba (China)-Hong Kong Polytechnic University Collaborative Research Project. The funders had no role in study design, data collection and analysis, decision to publish, or preparation of the manuscript. The remaining authors declare that the research was conducted in the absence of any commercial or financial relationships that could be construed as a potential conflict of interest.
